# The Impact of Impulsivity and Emotional Dysregulation on Comorbid Bipolar Disorder and Borderline Personality Disorder

**DOI:** 10.7759/cureus.9581

**Published:** 2020-08-05

**Authors:** Noha Eskander, Mina Emamy, Suhail M Saad-Omer, Farah Khan, Nusrat Jahan

**Affiliations:** 1 Psychiatry, California Institute of Behavioral Neurosciences & Psychology, Fairfield, USA; 2 Research, California Institute of Behavioral Neurosciences & Psychology, Fairfield, USA; 3 Medicine, California Institute of Behavioral Neurosciences & Psychology, Fairfield, USA; 4 Internal Medicine, California Institute of Behavioral Neurosciences & Psychology, Fairfield, USA

**Keywords:** bipolar disorders, borderline personality disorder, impulsivity, emotional dysregulation, psychiatry & mental health, mood and anxiety, child and adolescent psychiatry, global neurology, comorbid anxiety, psychology

## Abstract

The symptomatic overlap between borderline personality disorder (BPD) and bipolar disorder (BD) is a topic of scientific and academic debates. Emotional dysregulation and impulsivity are common features of both disorders. Several studies have shown that both BPD and BD lie on a spectrum; others have suggested that they are separate entities that coexist.

BPD is characterized by impulsive and dangerous behaviors such as driving recklessly, inappropriate sexual behavior, eating disorders, and substance abuse. BD, during a manic episode, is known for their impulsive and risk-taking behavior like hypersexuality, excessive spending, and substance abuse.

The current literature review aims to provide an overview of the impact of impulsivity and emotional dysregulation on comorbid bipolar disorder and borderline personality disorder. Our study results showed that patients with comorbid BPD and BD struggle with impulsive actions and have difficulty controlling their emotions. They are also highly susceptible to anxiety disorders like obsessive-compulsive disorder (OCD), post-traumatic stress disorder (PTSD), and somatoform disorders. Patients with comorbid BPD and BD struggle with severe psychosocial morbidity and an increased risk of suicide. In patients with only one disease, misdiagnosis is a common phenomenon due to the overlapping symptoms of BPD and BD.

## Introduction and background

Impulsivity is the tendency to act on an impulse without inhibitions and regard to the consequences. Impulsive actions are often poorly conceived, premature, and may lead to undesired outcomes [1]. Impulsivity consists of two components, one acting without inhibitions and consideration and second choosing immediate pleasure over long-term planning. Impulsivity is a major component in mental disorders like borderline personality disorder (BPD) and bipolar disorder (BD). Emotional dysregulation is the inability to refrain from reacting to provocative emotional stimuli. It constitutes experiences that interfere with goal-directed activity [2]. Individuals having BPD and BD struggle with emotional dysregulation.

Borderline personality disorder (BPD) is a mental illness characterized by long-term patterns of emotional instability, unstable relationships, low self-esteem, and a distorted self-image. Individuals with BPD are characterized by impulsive and dangerous behaviors such as driving recklessly, unsafe sexual behavior, eating disorders, and substance abuse [3]. Ten percent of individuals diagnosed with BPD commit suicide [4]. It was estimated that approximately one to two percent of the general population was susceptible to BPD, but a study in 2008 found a lifetime prevalence of 5.9% [5,6]. Women are three times more affected than men [7]. Women make more suicide attempts than men; however, men have a higher death rate due to suicide [4].

BD is a mental disorder characterized by periods of depression alternating with hypomania or mania. Depression is characterized by persistent low mood and energy, while the latter two have elevated mood and normal high to severely high energies, respectively. Mania contributes to impulsivity, hypersexuality, poor judgment, and a lack of inhibitions in BD. People with BD are at a high risk of committing suicide during depressive episodes due to difficulty in impulse control and emotional dysregulation [8]. The lifetime prevalence of BD in the general population is 2.4% [9].

The symptomatic overlap in BPD and BD is prone to academic debates and discussions and may lead to a possible misdiagnosis. Affective lability and impulsivity are common features of both disorders [10]. Some studies suggested that both BPD and BD lie on a spectrum; others suggested they are separate entities that coexist. Previous research studies reported that approximately 20% of BPD were also diagnosed with BD. Similarly, 10% of people with BD I and 20% with BD II were diagnosed with BPD [11].

The objective of this literature review was to evaluate how impulsivity and emotional dysregulation impact BPD and BD as separate and comorbid disorders. The objective was to increase our understanding of how patients with comorbid BPD and BD function socially, psychologically, and medically. Our study aimed to find out how the context of impulsive behavior differs in BPD compared to BD and how we can effectively diagnose patients with comorbid BPD and BD. In our literature review, we reviewed past research studies that have discussed Impulsive behavior and emotional dysregulation in BPD, BD, and comorbid BPD and BD. The goal was to draw outlines distinguishing common features like impulsivity and emotional dysregulation in these two disorders and avoid misdiagnosis.

## Review

Methods and results

Data was searched on PubMed using regular keywords: “Borderline Personality disorder (BPD),” “Bipolar disorder (BD),” “Impulsivity,” and “Emotional dysregulation.” Table 1 shows the search results of the regular keywords “Borderline Personality disorder (BPD)”, “Bipolar disorder (BD)”, “Impulsivity” and “Emotional dysregulation.”

**Table 1:** Regular keywords search result

Keywords	Database	Date	Number of results
Borderline Personality Disorder	Pubmed	6/19/2020	9674
Bipolar Disorder	Pubmed	6/19/2020	52,063
Impulsivity	Pubmed	6/19/2020	65,417
Emotional dysregulation	Pubmed	6/19/2020	4,379

Inclusion Criteria

1. All articles published within the last 15 years

2. Only human studies 

3. Articles published only in the English language 

Exclusion Criteria

1. All articles published before the last 15 years

2. Animal studies 

3. Articles published in languages other than English

Results

After applying the criteria and reviewing the articles, we chose only the relevant research studies for this literature review. A total of 35 articles were selected for our study to find out the impact of impulsivity and emotional dysregulation in comorbid bipolar disorder and borderline personality disorder.

Discussion

According to the results of our literature review, patients with comorbid BPD and BD struggle with impulsive behavior and emotional control more than patients with BPD or BD alone. Those patients show severe psychosocial morbidity and difficulty in behavioral adjustment.

Impulsivity

Impulsivity is the tendency to act quickly without enough thought or conscious judgment. The five traits that contribute to impulsive actions are positive and negative urgency, sensation seeking, low conscientiousness such as lack of planning, and lack of perseverance [12]. The Barratt Impulsiveness Scale (BIS) is the most common test used to measure personality-behavioral impulsive traits. It consists of three subscales: attention impulsiveness (inability to focus on a task at hand), motor impulsiveness (acting on the spur of the moment), and non-planning impulsiveness (acting on the present impulse with no regard to the future) [13]. The neurobiological findings for impulsive behavior include damage to the right inferior frontal gyrus in the prefrontal cortex (PFC), which leads to deficits in stop-signal necessary for inhibition [14]. Excitotoxic lesions in the nucleus accumbens core, lesions in the amygdala, and dorsum striatum result in preference of smaller and immediate rewards [15].

Emotional Dysregulation

It is a term used by mental health professionals to describe emotions that are poorly modulated and in an inappropriate response to a given stimulus. Emotional dysregulation includes anger outbursts, aggression toward others, and suicide threats. It interferes with daily functioning and social interactions and is commonly associated with impulsive actions. BPD is also known as an emotionally unstable personality disorder. Emotional dysregulation also presents in BD, which is a mood disorder [16].

Borderline Personality Disorder 

Individuals with BPD have difficulty regulating their emotions. Affective instability is a core feature of BPD. They often react intensely and in greater depth to situations with a slower return to the normal baseline. They react with rage instead of anger and shame instead of embarrassment. Individuals with BPD struggle with idealization and devaluation, feelings of fear of abandonment, emptiness, and a distorted self-image. Other impulsive behaviors include spending too much, driving recklessly, substance abuse, and binge eating [3]. Neuroimaging brain studies in BPD show a decrease in the size of the hippocampus and the amygdala, which are parts of the limbic system that are responsible for emotional control. This decrease in the size of the hippocampus and the amygdala might explain the feelings of aggression and impulsivity in BPD [17]. A meta-analytic study revealed that subjects with BPD had less activation of a network of regions that extended from the amygdala to the subgenual anterior cingulate and dorsolateral prefrontal cortex in response to processing negative emotions when compared to healthy control subjects [18]. The hypothalamic-pituitary axis (HPA axis) regulates cortisol release in response to stress. The HPA axis was found to be dysfunctional in BPD, therefore, resulting in continued high levels of cortisol [19]. This finding possibly explains the maladaptation to stress presented in BPD.

Bipolar Disorder 

Bipolar disorder is a mood disorder that can be classified into BD I, BD II, mixed affective state, cyclothymia, and rapid cycling. In bipolar I, at least one manic episode and at least one depressive episode are necessary for diagnosis. In bipolar II, one or more depressive episodes with one or more hypomanic episodes are necessary for diagnosis. In cyclothymia, there is a history of hypomanic episodes with periods of depression that do not meet the criteria of major depressive episodes [20]. Impulsivity is a core feature in BD, which varies during the mood cycles. Patients may express impulsivity in subsyndromal states as well. Impulsivity impairs the neurocognitive functioning in BD and increases the associated comorbidities like substance abuse disorder [21,22]. A cross-sectional study has found that certain aspects of impulsivity differ according to the polarity of the affective symptoms [23]. For example, motor impulsivity is associated with mania, while non-planning impulsivity is associated with depression. The link between impulsivity and depression is directly related to suicide [23]. The ventricular prefrontal cortex (vPFC), which regulates the limbic system, especially the amygdala, shows disrupted activity on functional MRI studies. This disruption possibly explains the labile mood and poor emotional regulation in BD. Manic and depressive episodes are also associated with dysfunction and decrease in the activity of the right and left parts of the vPFC, respectively [16]. Meta-analytic studies revealed that an increase in the activation in the ventral limbic brain regions in BD is responsible for emotional experiences and a decrease in the activation in the right hemisphere cortical structures that are related to cognition and emotional regulation [24]. There is evidence that there is an alteration in frontostriatal processing in manic patients, and this alteration is associated with preferring inferior immediate rewards compared to superior delayed rewards [25].

Borderline Personality Disorder and Bipolar Disorder

**Figure 1:**
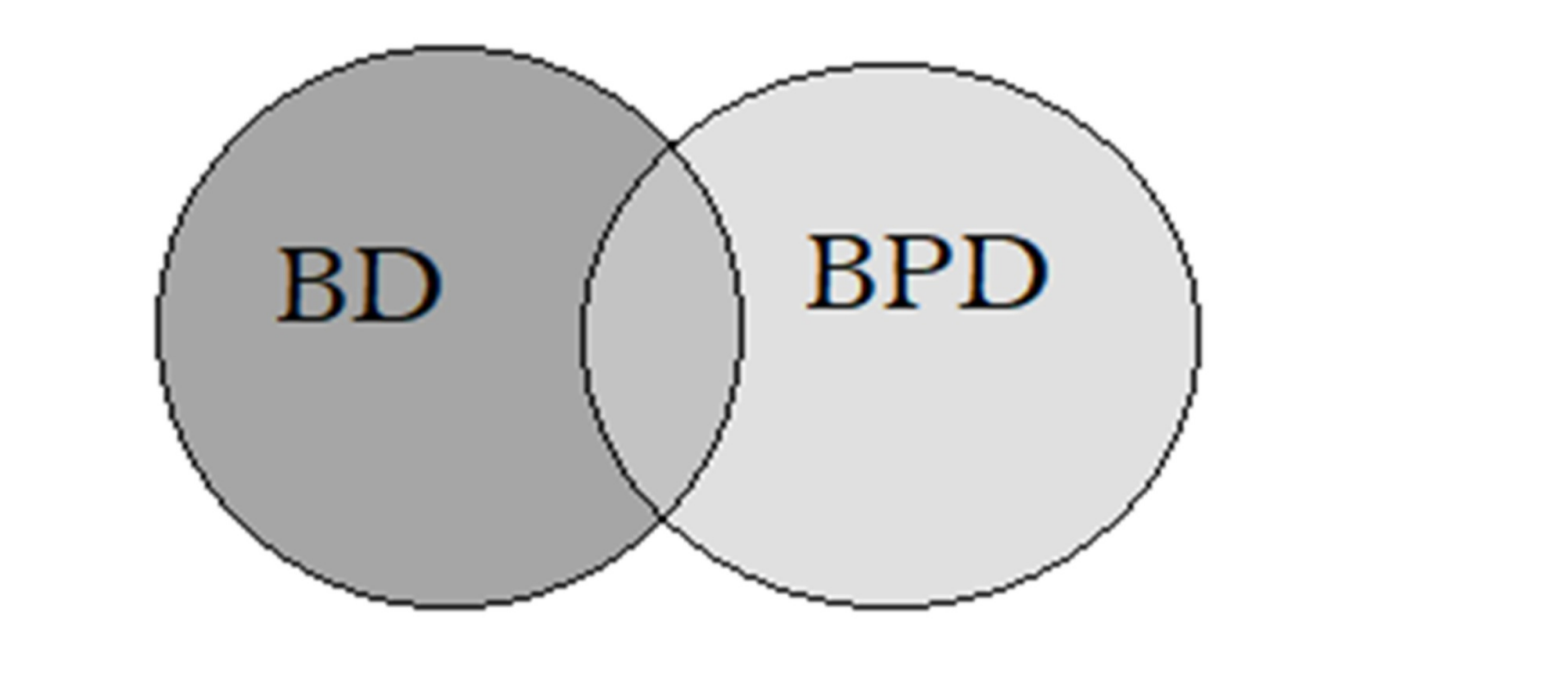
Symptom overlap and the indistinct diagnostic boundaries between BD and BPD BD: bipolar disorder, BPD: borderline personality disorder

**Figure 2:**
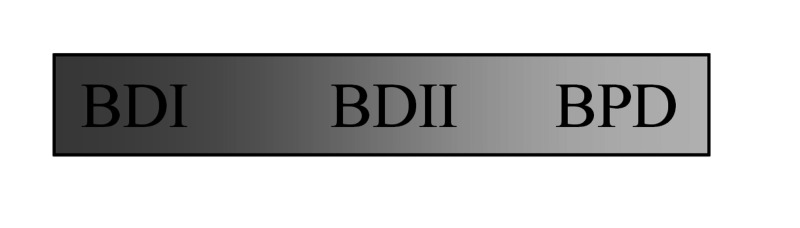
Possibility of BD and BPD being on a spectrum of one disorder BDI: bipolar I disorder, BDII: bipolar II disorder, BPD: borderline personality disorder

BPD and BD share common features like impulsivity and emotional dysregulation. The symptomatic overlap has resulted in a debate whether the diseases are separate with common features (Figure 1), part of the same spectrum (Figure 2), or separate disorders that are risk factors for one another [11,26]. Often it is difficult to draw distinct lines to separate BPD from BD leading to a dual diagnosis when only one disorder is present. Mood changes and affective lability in BPD could be perceived as bipolar mood changes, and consequently, BPD behavioral problems could be misunderstood as arising from symptoms of bipolar mood disorder. 

Some research studies have suggested that the occurrence of BD at an early age affects psychological development negatively and can cause BPD (the scar hypothesis). Correspondingly, the occurrence of BPD in adolescence can cause BD later in life (the vulnerability hypothesis). Both BPD and BD share common risk factors like the trait neuroticism [26]. Impulsivity, in general, is more common in BPD as compared to BD. According to a study by Leblanc et al., both BPD and BD patients were given three self-reported questionnaires: ALS (affective lability scale) to evaluate affective lability, AIM (affective intensity measure) to determine affective intensity, and UPPS (urgency, premeditation, perseverance, sensation seeking) to measure impulsivity in different dimensions. The results of the study showed that individuals with BPD scored significantly higher in affective lability and intensity and for the dimensions “lack of premeditation” and “lack of perseverance” compared to individuals with BD. Individuals with BD scored higher in “negative emergency” [27]. BPD and BD have different patterns of mood swings. Mood swings in BPD are usually triggered by events and last for hours or, rarely, days compared to BD, in which mood swings are random and last many days [28]. The male to female occurrence ratio in BD and BPD are 1:1 and 1:3, respectively [7]. The gender difference in BPD could be related to reporting biases. It was found that women who suffer from BPD are more likely to undertake more self-directed behaviors like self-harm, so they seek mental help more often than men [7].

Comorbid BPD and BD

Comorbid BPD and BD are associated with marked psychosocial disability. A study conducted by Frias and colleagues showed that BD patients with comorbid BPD have an earlier onset of mood symptoms when compared to those with BD alone [29]. They also have higher rates of hostility, substance abuse, and suicide. A study by Zimmerman found that patients with comorbid BPD and BD had three times higher diagnoses of axis one disorders than patients with BD alone, like obsessive-compulsive disorder (OCD), post-traumatic stress disorder (PTSD), and somatoform disorders, when compared individually. They reported more OCD than those with BPD alone. They were also at higher risks for depression, persistent unemployment, and hospitalization compared to BPD or BD patients alone [30]. Patients with comorbid BPD and BD are more impulsive and aggressive than patients with BPD and BD alone and, therefore, are at higher risk of suicide [31].

Childhood trauma is a common risk factor for both disorders. Previous studies suggested that childhood trauma modulates the expression of BD to rapid cycling type, which resembles the clinical features of BPD [32]. The misdiagnosis of comorbid BPD and BD is very common. Clinicians might miss on the diagnosis of one disorder due to the commonality in symptoms between the two [33]. The difficulties in anger control in BPD could be mistaken as irritability during a manic episode in BD [34]. The misdiagnosis could be as a result of a diagnosis bias or incomplete history in BD. For example, a patient with BD during a mood episode presenting with affective instability and relationship difficulty might be misdiagnosed with BPD. The misdiagnosis is especially common in BD II rather than in BD I due to the affective instability and the chronicity of depressive symptoms in BDII. Also, the mild elevation in mood in BD II may look like mood fluctuations BPD [33].

Fear of abandonment is a characteristic feature in patients with BPD. They are consistently anxious to be forsaken and left behind from people they are attached to. Patients with BD can also have this fear, but it is not a characteristic feature of the disorder. The euphoric mood is a main aspect in BD during the manic episodes which does not characterize individuals with BPD. Most clinicians have a tendency to diagnose BPD patients with BD because BD is supported by past extensive research studies. BD, in general, is less socially stigmatized as a mental health problem compared to BPD so it is easier for clinicians to explain the disorder to the patient. A detailed past medical and social history should be documented in patients presented with the symptomatic overlap in BD and BPD, to determine whether the functional impairment is restricted to mood episodes or is a continued rigid pattern of thinking and behaving that belong to a personality disorder [35].

Study Limitation

Our study is based on reviewing research studies in the last 15 years, so possibly we missed other important contributions from studies before 2005. We did not perform a systematic review in our study and no quality assessment of the selected research studies was done.

## Conclusions

Emotional dysregulation and impulsivity are core features for both BPD and BD. Impulsivity is a more prominent feature for BPD compared to BD. Childhood trauma is a common risk factor for both disorders and there is a strong underlying neurobiological evidence for impulsivity and emotional dysregulation in both. Individuals with comorbid BPD and BD show significant psychosocial morbidity and an increase in suicide risk. Misdiagnosis is common in comorbid BPD and BD due to the similarities in the presentation of both conditions. 

A careful review of the patient’s history is crucial for clinicians to properly distinguish between BPD and BD and to make the right diagnosis. Special attention and follow up should be directed to the patients with comorbid BPD and BD regarding the increase in suicide risk. Newly modified screening tools are needed to screen for BPD in patients with mood disorders, and new research studies are essential to investigate the risk factors, the genetic aspect, and the prognosis of patients with comorbid BPD and BD.
